# Potentiodynamic Fabrication of Aromatic Diamine Linkers on Electrochemically Reduced Graphene Oxide Surface for Environmental Pollutant Nitrobenzene Monitoring

**DOI:** 10.3390/bios16010033

**Published:** 2026-01-01

**Authors:** Karmegam Muthukrishnan, Venkatachalam Vinothkumar, Mathur Gopalakrishnan Sethuraman, Tae Hyun Kim

**Affiliations:** 1 Centre for Smart Energy Systems, Chennai Institute of Technology, Chennai 600069, India; muthukrishnank.chem@citchennai.net; 2 Department of Chemistry, The Gandhigram Rural Institute-Deemed to be University, Gandhigram 624302, India; 3 Korea Native Animal Resources Utilization Convergence Research Institute, Soonchunhyang University, Asan 31538, Republic of Korea; vinothvr66@gmail.com; 4 Department of Chemistry, Soonchunhyang University, Asan 31538, Republic of Korea

**Keywords:** self-assembled monolayers, aromatic diamines, electrochemically reduced graphene oxide, nitrobenzene sensor, environmental sample

## Abstract

The structure of self-assembled monolayers (SAMs) greatly influences electrochemical interface behavior. This study systematically examines how positional isomers of aromatic diamines (ADMs) assemble on a glassy carbon (GC) electrode and how such ordering affects the attachment and performance of electrochemically reduced graphene oxide (ERGO). SAMs of ortho-, meta-, and para-phenylenediamine (o-PDA, m-PDA, and p-PDA) were fabricated on GC and characterized using atomic force microscopy (AFM) and Raman spectroscopy. Among them, GC/p-PDA exhibited the most compact and homogeneous interfacial structure. ERGO was subsequently immobilized through the free amine functionalities of the SAM, as confirmed by attenuated total reflectance–Fourier transform infrared spectroscopy (ATR-FTIR), X-ray photoelectron spectroscopy (XPS), and cyclic voltammetry (CV). Strong covalent coupling and electrostatic interactions between the positively charged ERGO and terminal amines enabled stable attachment. Under optimized conditions, the modified GC/p-PDA/ERGO electrode demonstrated exceptional electrocatalytic activity toward nitrobenzene (NBz) reduction, achieving a high sensitivity of 1410 μA mM^−1^ cm^−2^ and a low detection limit of 0.040 μM. In addition, this sensor displayed outstanding anti-interference capability, stability, and recovery in a water sample. These results establish GC/p-PDA/ERGO sensor as a robust and efficient electrocatalytically active interface for nitroaromatic pollutants detection and sustainable environmental monitoring.

## 1. Introduction

Graphene and related materials hold significant value for next-generation electrochemical sensors, as their physicochemical properties offer an unprecedented combination in previous materials [1]. A two-dimensional sheet-like structure can provide a broad surface for molecular correlations, while a consistent π-electron lattice secures remarkable electrical and mechanical properties [2]. To further make them suitable for long-lasting electrochemical interfaces, intrinsic chemical inertness is added [2]. Among the various forms available, graphene oxide (GO) is a versatile and scalable starting material that can be transformed through chemical or electrochemical reduction into electrochemically reduced graphene oxide (ERGO) [3,4]. This reconstruction enhances charge-carrier mobility while leaving some oxygen-bearing groups stable [5]. As a result of these persistent functionalities as well as improved conductivity, ERGO is particularly effective for the integration of catalytic sensing molecules as well as the promotion of charge transfer [6]. Consequently, ERGO-layered electrodes have become a backbone in environmental surveillance, biosensing, and other electrochemical evaluation applications [6].

Numerous techniques have been explored to tether graphene and its derivatives to electrode surfaces. These include creating self-assembled monolayers (SAMs) [7], electrodeposition [8], simple drop coating [9], sequential layer assembly [10], and vapor phase deposition [11]. Among these, the SAMs approach is particularly attractive because it allows precise control at the molecular scale, yields highly uniform films, and is relatively straightforward to carry out [7]. The formation of a monolayer in this way promotes the orderly, reproducible attachment of graphene to the electrode, which enhances the stability of the interface and improves the performance of the sensor [7]. Traditionally, much attention has been given to monolayers of alkanethiols on gold electrodes, where the sulfur ends of the molecules chemisorb strongly to the metal, creating densely packed and well-ordered films [12]. Despite their regular structure, these thiol-based layers are susceptible to oxidation, which compromises their long-term stability and drives up the cost of fabrication [13].

Molecules bearing terminal amine groups, especially those with two amine units, provide a resilient way of modifying carbon-based substrates such as glassy carbon (GC), an electrode [14]. These compounds bond to the carbon surface through nucleophilic additions similar to a Michael reaction, producing dense and chemically stable coatings [15]. The presence of two amine groups not only improves adhesion but also provides binding sites for attaching nanostructures such as graphene, metal nanoparticles, and hybrid metal-nanocomposite materials [15,16]. Compared with sulfur-based SAMs, diamine-derived surfaces are more durable, chemically robust, and reproducible, and they can be further functionalized with relative ease [16]. Such characteristics make diamine SAMs appealing for creating durable and cost-effective electrochemical sensors. Monolayers made from aliphatic diamines (ADAs) are already widely used on GC electrodes, after which various metal and carbon nanostructures are incorporated for sensing [15,16]. In contrast, the formation of similar surfaces from aromatic diamines (ADMs) on the GC-electrode has scarcely been explored. In this study, for the first time, the study demonstrates that ADMs can form ordered films on GC-electrode and that these layers can be functionalized with ERGO to enable electrochemical detection of nitrobenzene (NBz).

Ensuring access to safe drinking water is an urgent global issue, yet the same industrial growth that drives economic development often results in polluted waterways [17]. NBz, an intermediate in the manufacture of aniline, explosives, dyes, pesticides, rubber additives, and pharmaceuticals, is one of the most harmful pollutants [18]. Classified as a persistent organic pollutant, it is highly stable in the environment and can accumulate in living organisms, posing serious risks to human health and ecological balance over the long term [19,20]. Furthermore, the World Health Organization (WHO) advises against above 2 mg/L of NBz in water. Considering these matters, various conventional detection techniques, such as gas chromatography–mass spectrometry [21], fluorescence measurements [22], flow injection analysis [23], chemiluminescence [24], and colorimetric assays [25] were employed for NBz reduction. But all these techniques are expensive, labor-intensive, time-consuming, and require complicated instruments. They also show poor selectivity and a higher detection limit. Hence, there is an urgent need for portable, highly selective, and sensitive detection methods in water matrices. Electrochemical sensors meet this need by offering fast, selective measurements with slight sample preparation, and they function effectively over wide concentration ranges and under varied field conditions to provide real-time data on NBz pollution [15,16]. We recently explored ADAs of SAMs with ERGO for NBz detection, demonstrating the remarkable electrochemical activity [15]. Subsequently, in this work, we developed different ADMs of SAMs grafted onto ERGO towards electrochemical sensing of NBz. The p-PDA/ERGO hybrid not only improves dispersibility, chemical stability, conductivity, and surface characteristics, but it also provides a large number of active sites, which increases electron transport to NBz. As a result, the GC/p-PDA/ERGO electrode has a much higher reduction current towards NBz than bare GC, GC/p-PDA, GC/ERGO, GC/o-PDA/ERGO, and GC/m-PDA/ERGO electrodes. Furthermore, the GC/p-PDA/ERGO sensor demonstrated outstanding electrochemical performance for NBz detection, including a low detection limit and ultra-high sensitivity in amperometry analysis.

The purpose of this study is therefore to determine whether ADM molecules can assemble into homogeneous, chemically robust monolayers and whether these layers can be used for electrochemical sensing. The three structural isomers of ortho-phenylenediamine (o-PDA), meta-phenylenediamine (m-PDA), and para-phenylenediamine (p-PDA) have different positional arrangements of their amino groups, which lead to distinct adsorption patterns that affect monolayer packing, graphene attachment, and sensor performance. The results reveal that monolayers derived from these molecules strongly hinder electron transfer, reflecting the formation of dense and well-ordered films. It is possible to gain a deeper understanding of the surface chemistry and charge transfer behavior of these systems by systematically studying how each isomer of SAMs on the GC electrode interacts with ERGO and influences the electrochemical reduction of NBz. These insights can be used to develop a robust and highly sensitive monolayer-based electrochemical sensor for environmental monitoring.

## 2. Materials and Methods

### 2.1. Materials

Hexaammineruthenium (III) chloride ([Ru(NH_3_)_6_]Cl_3_), para-phenylenediamine (p-PDA), and meta-phenylenediamine (m-PDA) were procured from Sigma-Aldrich. Ortho-phenylenediamine (o-PDA) was purchased from Loba Chemie Pvt. Ltd. (Mumbai, India). Graphite powder, potassium permanganate (KMnO_4_), hydrogen peroxide (H_2_O_2_), nitrobenzene, and sodium nitrate (NaNO_3_) were obtained from Merck (Mumbai, India). Sulfuric acid (H_2_SO_4_), sodium dihydrogen phosphate (NaH_2_PO_4_), disodium hydrogen phosphate (Na_2_HPO_4_), hydrochloric acid (HCl), and ethanol were supplied by Emplura. Thermo Fisher Scientific provided the glassy carbon (GC) plates. All the above chemicals were used without additional purification. Double-distilled water was used to prepare the phosphate-buffered solution (PBS), which served as the supporting electrolyte for the electrochemical studies.

### 2.2. Instrumentation

Electrochemical characterization, including cyclic voltammetry (CV) and electrochemical impedance spectroscopy (EIS), was carried out using a CHI 643B electrochemical workstation (CH Instruments, Austin, TX, USA). A standard three-electrode setup was used, consisting of a GC working electrode, a platinum wire counter electrode, and a reference electrode of Ag/AgCl (saturated NaCl). An ESCA Microprobe PHI 5000 VersaProbe system (ULVAC-PHI, Chigasaki, Japan) was used to obtain XPS data, with binding energy reproducibility kept within ±0.10 eV. UV–Vis absorption spectra were recorded on a JASCO V-650 spectrophotometer. The surface morphology of the modified electrodes was visualized using a TESCAN VEGA3 scanning electron microscope (TESCAN, Brno, Czech Republic). Atomic force microscopy (AFM) images were captured in tapping and 3D models with a 5500 Series multimode scanning probe microscope (Agilent Technologies, Santa Clara, CA, USA). In addition to collecting Raman spectra, ATR-FTIR spectra were collected using a horizontal ZnSe crystal fitted to a JASCO FT/IR-460 Plus spectrometer (Jasco Deutschland GmbH, Hessen, Germany).

### 2.3. Preparation of GO

GO was prepared using a modified version of Hummers’ procedure [3]. In brief, 0.5 g of graphite and 0.25 g of sodium nitrate were dispersed in 12 mL of concentrated sulfuric acid under vigorous stirring while the mixture was kept in an ice bath. Potassium permanganate was gradually introduced, keeping the temperature below 20 °C. After the oxidant was added, the slurry was allowed to warm to approximately 35 °C and stirred at that temperature for 30 min. This was done to complete the oxidation. After adding 23 mL of double-distilled water dropwise, the temperature climbed exothermically to around 98 °C, which was maintained for 15 min with external heating. Afterward, 72 mL of water was added, followed by careful addition of 0.5 mL of 30% hydrogen peroxide until the suspension became bright yellow. The mixture was cooled, repeatedly washed with dilute hydrochloric acid (0.1 M) and double-distilled water to remove residual metal ions, and dried to obtain brownish-yellow GO sheets.

### 2.4. Formation of ADM Monolayers on GC-Electrode

GC-electrodes (3 mm in diameter) were sequentially polished with alumina slurries of 0.3 µm and 0.05 µm to achieve a mirror finish, rinsed thoroughly with double-distilled water, and ultrasonicated to remove any remaining abrasives. For monolayer formation, the cleaned GC-electrodes were immersed for 9 h at room temperature in separate 10 mM ethanolic solutions of o-PDA, m-PDA, and p-PDA. The amine groups react with the carbon surface through a nucleophilic addition mechanism analogous to the Michael reaction [15,26]. As a result, ADM monolayers are assembled in an ordered manner. The resulting modified electrodes were designated GC/o-PDA, GC/m-PDA, and GC/p-PDA electrodes. Surface composition and film quality were assessed by XPS, ATR-FTIR, EIS, and CV.

### 2.5. Self-Assembly of GO and Conversion to ERGO Using ADM Linkers

**Scheme 1:**
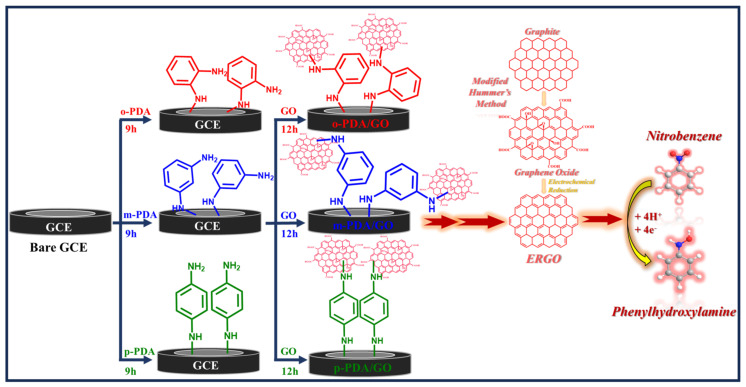
Schematic representation of the stepwise fabrication of ADM-modified GC-electrodes (GC/ADMs/ERGO) and their application in NBz electrochemical sensing.

After modification with ADMs, the electrodes were thoroughly rinsed with double-distilled water to remove any loosely adsorbed molecules and then immersed in a 1 mg mL^−1^ suspension of exfoliated GO for 12 h. To ensure proper dispersion, the GO suspension was made in double-distilled water and sonicated for 45 min before use. Depending on which diamine was used, the resulting assemblies were termed GC/o-PDA/GO, GC/m-PDA/GO, and GC/p-PDA/GO electrodes. Attachment of GO to the diamine-modified surfaces occurred through a combination of Michel nucleophilic addition and electrostatic attraction [15,26]. The acidic GO dispersion (pH ≈ 2.5) protonated the terminal amines, giving them a positive charge and promoting electrostatic interaction with the negatively charged GO sheets [3,27]. These interactions produced strong and uniform coatings. After immobilization, the GO films were electrochemically reduced in PBS (pH 7.2) to produce ERGO. The resulting electrodes GC/o-PDA/ERGO, GC/m-PDA/ERGO, and GC/p-PDA/ERGO were used in electrochemical experiments (Scheme 1). For comparison, a control electrode (GC/ERGO) was prepared by depositing GO directly onto an unmodified GC electrode and reducing it under the same conditions.

## 3. Results and Discussion

### 3.1. Formation Mechanism of ADM Monolayers on the GC Surface

**Scheme 2:**
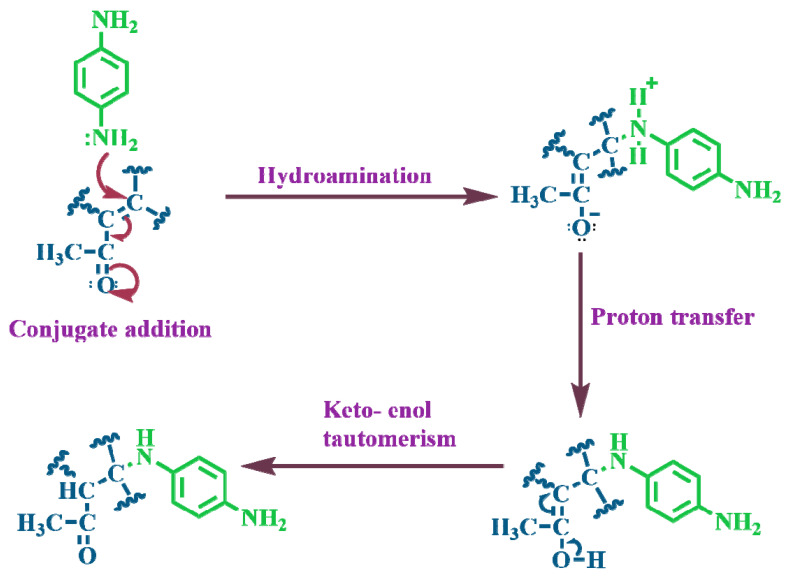
Schematic representation of the proposed pathway describing the spontaneous interaction between surface amine functionalities and the carbon electrode surface.

The phenylenediamine isomers studied here, o-PDA, m-PDA, and p-PDA, attach to the GC-surface through a nucleophilic addition mechanism reminiscent of a Michael reaction [26], shown in Scheme 2. Under the experimental conditions, the amine terminals are activated and become strong nucleophiles [26]. Although benzene rings typically undergo electrophilic substitution, the nitrogen atoms in the amino groups possess lone pairs that can attack electrophilic β-carbons on mildly oxidized carbon surfaces, forming C–N bonds [15,28]. These reactive sites on the carbon are often α,β-unsaturated carbonyls or carboxyl groups; resonance stabilization from nearby electron-withdrawing groups facilitates bond formation, and subsequent proton transfer neutralizes the charge, yielding a covalently bonded monolayer [29]. Such films are chemically robust and resist desorption or oxidation far better than assemblies based on Au–S bonds or simple π–π stacking [26,29]. The strength of the resulting C–N (or occasional C–O) linkage imparts exceptional stability under extended potential cycling and elevated temperatures [26]. Moreover, this chemistry produces uniform, well-packed monolayers under mild conditions and can be applied to a variety of carbon substrates, including graphene and carbon nanomaterials, as well as to the immobilization of metal and hybrid nanostructures [15,16]. Compared with the more expensive and oxidation-prone thiol-based monolayers, ADM coatings offer a durable and cost-effective route for functionalizing electrodes for sensing, catalysis, energy storage, and protective coatings [30,31].

### 3.2. Modification of ERGO-Fabricated Electrodes

GO was attached to GC-electrodes using these diamine linkers. UV–visible spectra of the synthesized GO showed a prominent absorption near 236 nm due to π–π* transitions in C=C bonds and a shoulder around 305 nm from n–π* transitions of oxygenated groups, confirming its successful preparation [15] (Figure S1). When GO is functionalized onto a bare GC electrode, poor adhesion and sluggish charge transfer lead to unstable films and a declining current response during electrochemical cycling [15,32]. The high density of oxygen groups also disrupts the conjugated π system, reducing conductivity and active surface area. Introducing ADMs as molecular linkers circumvents these issues. They improve the dispersion of GO, strengthen interfacial bonding, and facilitate electron transfer, which results in more stable electrodes and higher currents. The GO suspension was acidic (pH ≈ 2.5), so the amine groups of the linkers were protonated, promoting electrostatic attraction to the negatively charged GO sheets and yielding a uniform film. The GO layer was then electrically reduced by changing the voltage between 0 and −1.2 V in PBS for 15 cycles, as shown in Figure S2. The ERGO-modified electrode potentials and reduction currents are displayed in Table S1. During the initial cycles, cathodic peaks appeared at approximately −0.953 V for GC/ERGO, −0.943 V for GC/o-PDA/ERGO, −0.945 V for GC/m-PDA/ERGO, and −0.925 V for GC/p-PDA/ERGO electrodes, corresponding to the removal of oxygenated groups [3,27,33]. These peaks diminished in subsequent scans as the sp^2^ carbon network was restored. A small feature around −0.5 V was also observed, which may be associated with partial reduction of protonated amines and suggests subtle differences among the linkers [3,27,33]. Among the electrodes studied, the p-PDA-modified surface delivered the largest current, likely because its para substitution allows closer packing of the molecules, stronger π–π interactions with the GO sheets, and a more conductive interface. The o-PDA coating, hindered by steric effects at the ortho positions, probably forms less ordered films with weaker GO attachment and slower electron transfer, while m-PDA shows intermediate behavior [34,35,36,37]. These observations are consistent with previous reports that the reduction potential of GO shifts to more negative values under alkaline conditions, highlighting the influence of protons on the reduction process [38].

GO + a H^+^ + b e- → ERGO + c H_2_O + d O_2_ (1)

From these findings, a reduction mechanism can be proposed in which the relative contributions of hydroxyl, carboxyl, carbonyl, and epoxide groups are represented by coefficients in the overall reaction [38,39,40].

### 3.3. Investigation of Surface Structural Chemistry Through ATR-FTIR and Raman Spectroscopy

**Figure 1:**
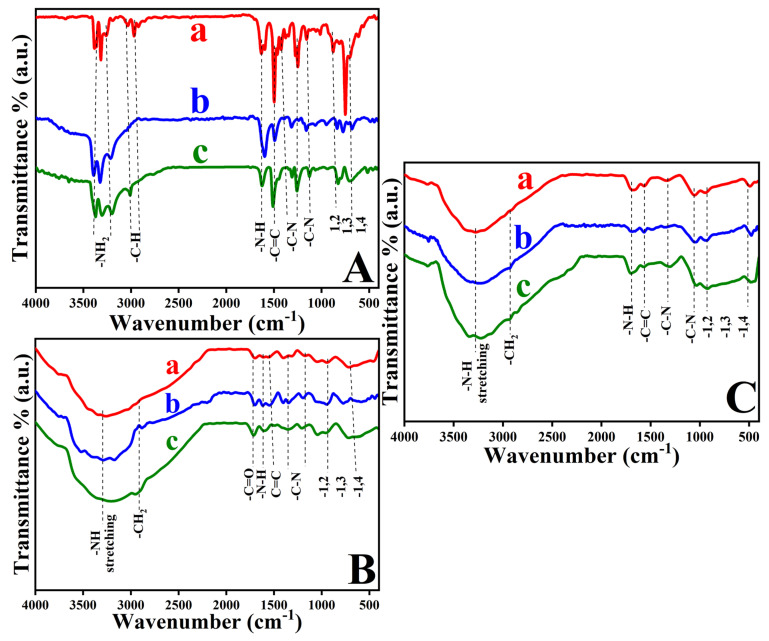
ATR–FTIR spectra of GC substrates modified with (**A**) o-PDA (a), m-PDA (b), and p-PDA (c); (**B**) o-PDA/GO (a), m-PDA/GO (b), and p-PDA/GO (c); and (**C**) o-PDA/ERGO (a), m-PDA/ERGO (b), and p-PDA/ERGO (c).

The sequential modification of GC substrates first by assembling ADMs and then by self-assembling GO and reducing ERGO was tracked by ATR-FTIR and Raman spectroscopy. Figure 1 shows ATR-FTIR spectra that highlight the chemical changes at each step. In the spectra of GC-substrates coated with o-PDA, m-PDA, and p-PDA (Figure 1A), broad bands between roughly (3200–3400 cm^−1^) correspond to the symmetric and asymmetric N–H stretching modes of primary amine groups, confirming that the diamine molecules have been anchored. Bending (scissoring) vibrations of the amines near 1610–1626 cm^−1^ indicate that these functionalities remain intact after self-assembly [41,42]. The aromatic framework is evidenced by C–H stretching bands around 2900–3030 cm^−1^ and C=C skeletal vibrations between 1495 and 1595 cm^−1^. Out-of-plane C–H bending modes that reveal the substitution pattern on the benzene ring were also observed [42]. Approximately 750–770 cm^−1^ for the ortho isomer (1,2-disubstitution), 780–810 cm^−1^ for the meta isomer (1,3-disubstitution), and 830–860 cm^−1^ for the para isomer (1,4-disubstitution) [41,42,43,44]. Together, these spectral features demonstrate that each ADM isomer has been successfully functionalized onto the GC surface. A summary of the ATR-FTIR data for the GC/o-PDA, GC/m-PDA, and GC/p-PDA assemblies is provided in Table S2.

Once GO was deposited onto the ADM-modified GC-substrates (Figure 1B), the ATR-FTIR spectra changed markedly. A broad absorption between about (3106–3456 cm^−1^) appeared, arising from the O–H stretching vibrations of hydroxyl groups in the GO [3]. This band partially overlaps the N–H region of the underlying diamine layer. The fact that the N–H stretching and bending features persisted shows that the amine groups remained chemically active beneath the GO immobilization. A pronounced carbonyl stretch at 1707–1720 cm^−1^ confirmed the presence of carboxylic groups in the oxide, and strong C–O bands between 1050 and 1250 cm^−1^ were attributed to epoxy and alkoxy functionalities [15]. The continued presence of aromatic C=C and out-of-plane C–H bands (700–900 cm^−1^) indicated that the diamine interlayer was stable during GO functionalization [15]. These interactions are summarized in Table S3.

The ATR-FTIR spectra changed after electrochemical reduction of GO to ERGO (Figure 1C). The broad O–H band (≈3200–3600 cm^−1^) and the carbonyl peak near 1720 cm^−1^ diminished markedly, indicating the removal of hydroxyl and carboxyl groups [3,27]. Moreover, the C–O stretching region (1050–1250 cm^−1^) lost substantially of its intensity, reflecting the loss of epoxy and alkoxy groups. There were still N–H stretching and bending vibrations, though they were weaker than before, suggesting the amine groups of the diamine linkers were partially retained and may interact weakly with the restored graphene surface [15]. A sharper aromatic C=C stretch at 1580–1600 cm^−1^ signalled re-establishment of the sp^2^-conjugated carbon network characteristic of ERGO [27]. The persistence of substitution-specific out-of-plane C–H bands (700–900 cm^−1^) further confirmed that the diamine interlayer remained chemically and structurally intact through the reduction. Together, these ATR-FTIR results corroborate the sequential formation of the diamine monolayer, anchoring of GO, and its conversion to ERGO. It is the presence of N–H vibrations at every stage that underscores the chemical resiliency of diamine linkers, whose amine groups continue to bridge the molecular layer and graphite surface in the final GC-surface [15]. Additional evidence of the interaction between the ADMs/ERGO is provided in Table S4.

**Figure 2:**
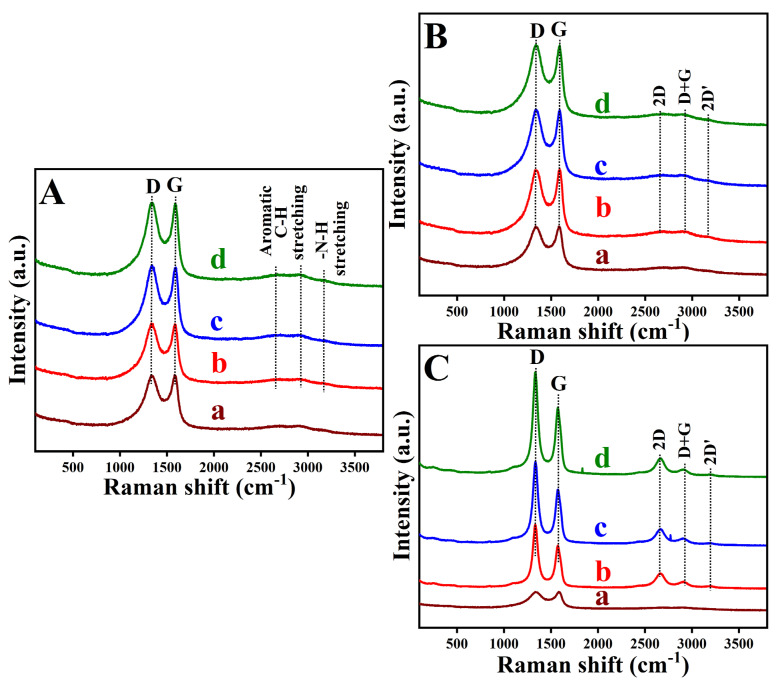
Raman spectra recorded for (**A**) unmodified and ADM-functionalized GC surfaces: (a) unmodified GC, (b) GC/o-PDA, (c) GC/m-PDA, and (d) GC/p-PDA; (**B**) GO-modified, and (**C**) ERGO-modified substrates corresponding to (a) bare GC, (b) GC/o-PDA, (c) GC/m-PDA, and (d) GC/p-PDA surfaces.

Raman spectroscopy was used to trace how the structure of the GC-surface changed during the modification process (Figure 2). On electrodes coated with the ADMs, the disorder-sensitive D band and the graphitic G band served as indicators of lattice defects and ordering (Figure 2A). In an unmodified GC-surface, the D band appears at about 1326 cm^−1^. After attachment of the ortho isomer, it shifts slightly to 1329 cm^−1^, and for the meta and para isomers, it moves to around 1338 cm^−1^ with an accompanying increase in intensity [45,46]. These shifts and intensity changes signify that attaching the diamines introduces new defect sites and perturbs the structure. The G band, which is at 1582 cm^−1^ on bare GC, moves to 1584 cm^−1^ for o-PDA, 1588 cm^−1^ for m-PDA, and 1590 cm^−1^ for p-PDA, and becomes progressively stronger, indicating strong electronic coupling between the substrate and the diamine layer [45,46,47]. Variations in the ratio of D- to G-band intensities reflect differences in how densely each isomer covers and functionalizes the surface. A weak C–H stretching band near 2660 cm^−1^ on unmodified GC shifts to 2670 cm^−1^ for o-PDA, 2673.1 cm^−1^ for m-PDA, and 2675.3 cm^−1^ for p-PDA, and its intensity increases, consistent with a Michael-type nucleophilic addition at the carbon surface [26]. In the region of symmetric aromatic C–H vibrations (~2895 cm^−1^), a peak at 2880.5 cm^−1^ on the bare GC moves to 2910.3 cm^−1^ (o-PDA), 2912.5 cm^−1^ (m-PDA), and 2920 cm^−1^ (p-PDA), confirming incorporation of aromatic groups. The para isomer produces the most intense signal, suggesting a densely packed monolayer. A small feature near 3150.9 cm^−1^, attributed to adsorbed moisture or residual oxygen on the bare GC substrate, shifts slightly upward to 3160.7 cm^−1^, 3162.6 cm^−1^, and 3163.2 cm^−1^ for the o-PDA, m-PDA, and p-PDA-isomers, respectively, reflecting N–H stretching and further confirming diamine attachment [48,49,50].

When the surfaces were coated with GO, the Raman spectra changed. The D band broadened and weakened around 1340 cm^−1^ due to oxygen-induced defects, and the G band at about 1580 cm^−1^ remained moderate, reflecting the presence of partly intact sp^2^ domains (Figure 2B). After electrochemical reduction, the ERGO films showed sharper D bands (1325–1336 cm^−1^) and G bands that shifted to lower wavenumbers (1565–1579 cm^−1^) and grew more intense, indicating partial restoration of the conjugated carbon network accompanied by an increase in defect density [45,46,47]. The I_D_/I_G_ band intensity ratio increased from roughly (0.99–1.0) for the GO-functionalized substrates to about 1.30–1.37 for the ERGO surfaces, consistent with defect creation and partial graphitization. Among the three diamine linkers, the para isomer yielded the most ordered ERGO coating, likely because its geometry allows linear packing, stronger π–π interactions, and greater charge delocalization.

The greater D-band intensity and downshifted G band in the ERGO films (Figure 2C) confirm structural reorganization and the removal of oxygen groups. A pronounced 2D band at 2655–2668 cm^−1^ in the reduced samples indicates improved graphitic order, whereas the GO-modified surfaces displayed weaker 2D bands consistent with oxygen-disrupted few-layer structures [46]. The appearance of a D + G combination band around 2905–2915 cm^−1^ signifies lattice distortion in GO, and its increased intensity after reduction suggests partial recovery of graphitic domains. Similarly, a 2D′ overtone band at 3192–3204 cm^−1^, faint in the GO, becomes more prominent after reduction, signaling the re-establishment of π–π conjugation. Taken together, the Raman data depict a clear progression from pristine GC through diamine modification and GO deposition to the partially graphitized ERGO surface. The electrochemical reduction primarily alters the defect landscape rather than producing fully crystalline graphene, and the diamine interlayers remain intact while enabling a controlled reconstruction of the surface [51].

On GO-fabricated surfaces, the Raman D band around 1340 cm^−1^ appeared broadened and of lower intensity, a clear signature of lattice defects introduced by oxygen. Furthermore, a moderately intense G band was observed near 1580 cm^−1^, suggesting that a few sp^2^-hybridized domains were still present in the oxidized material. After electrochemical reduction to form ERGO, the GC surface’s Raman spectra showed a sharper D band (shifted to ~1325–1336 cm^−1^) and a more intense, downshifted G band (~1568–1579 cm^−1^). These spectral changes confirm a partial restoration of the conjugated sp^2^ carbon network along with an increase in defect density during reduction. Consistently, the I_D_/I_G_ intensity ratio increased from about 0.99–1.00 in the GO-modified surfaces to roughly 1.30–1.37 in the ERGO surfaces, indicative of additional defect formation and partial graphitization as a result of the reduction process. Among the different diamine isomers tested, the ERGO derived from (p-PDA) exhibited the most ordered carbon structure [47,51]. Due to para-substituted molecular geometry, linear packing of molecules and stronger stacking interactions are achieved, therefore allowing the ERGO surface to be more effectively charged.

Further evidence of structural reorganization is provided by newly formed Raman bands upon reduction. As shown in Figure 2C, oxygen-containing functional groups were removed with ERGO more pronounced D and downshifted G bands. In the ERGO, a strong 2D band emerges at ~2655–2668 cm^−1^, which is clear evidence of improved graphitic ordering in the material. In contrast, the GO-modified electrodes show only a very weak 2D band, suggesting that the presence of oxygen had disrupted the formation of well-ordered few-layer graphene structures. Furthermore, a combined D + G band (appearing around 2905–2915 cm^−1^) is present in the spectra [47]. In GO, this D + G band signifies lattice distortions, and the fact that it becomes much more intense after reduction implies that some graphitic domains have been partially restored in ERGO. Likewise, the 2D′ overtone band (~3192–3204 cm^−1^), which is barely visible in GO, becomes distinctly pronounced in the ERGO. This enhancement of the 2D′ band confirms that extended π–π conjugation (the hallmark of graphitic carbon networks) has been re-established to a significant extent in the reduced material [45,46,47,51].

Raman spectra of bare GC, GC/ADMs, GC/ADMs/GO, and GC/ADMs/ERGO demonstrate GC surface modification at each stage. This progression indicates that the electrochemical reduction step mainly alters the defect landscape of the carbon material, introducing and reorganizing defects rather than producing a fully graphitic (defect-free) structure. It also shows that the ADM interlayers play a protective role, preserving the GC surface’s overall structural integrity while still allowing for a controlled reconstruction of the surface during the reduction process.

### 3.4. Surface Chemical Investigation by XPS

**Figure 3:**
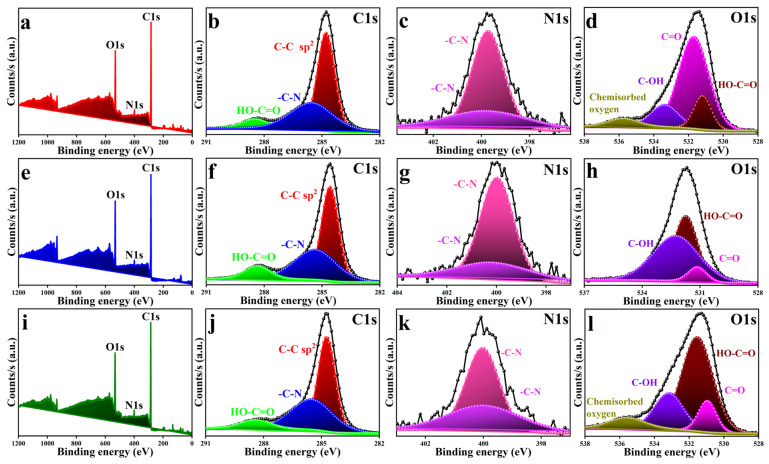
XPS spectra of the ADM/ERGO-modified GC substrates: (**a**), (**e**), and (**i**) show the survey spectra; (**b**), (**f**), and (**j**) depict the high-resolution C 1s spectra; (**c**), (**g**), and (**k**) correspond to the N 1s spectra; and (**d**), (**h**), and (**l**) represent the O 1s spectra for GC/o-PDA/ERGO, GC/m-PDA/ERGO, and GC/p-PDA/ERGO, respectively.

XPS, a technique widely used to probe surface composition and bonding, was used to characterize the GC substrates modified with o-PDA, m-PDA, and p-PDA on ERGO, as shown in Figure 3 (GC/o-PDA/ERGO, GC/m-PDA/ERGO, and GC/p-PDA/ERGO). The broad survey scans for each sample shown in Figure 3a,e,i are strong C 1s, N 1s, and O 1s peaks, indicating that the ADM linkers were attached to the GC substrate and that GO had been immobilized and subsequently reduced. Figure 3b,f,j shows the analysis of the high-resolution C1s region, which revealed three major components on all three GC substrates. The dominant peak at ~284.5–284.7 eV is characteristic of sp^2^ carbon (C–C/C=C) in the GC and ERGO backbone. The sp^2^-hybridised carbon in graphite or graphene typically appears between 284.0 and 284.5 eV. Among the other features near 285.3–285.5 eV were C–N bonds, which are tethered to the carbon framework by amine groups. The amine C–N bonds typically exhibit binding energies of around 286 eV. The third component at approximately 288.3–288.4 eV was assigned to carbonyl or carboxyl (O–C=O) groups located at the edges of the ERGO sheets. Carbonyl/carboxyl carbon generally appears near 288–289 eV [3,27,52,53]. The consistent presence of these peaks across the o-PDA, m-PDA, and p-PDA isomer series confirms strong chemical interactions between the diamine linkers and the graphitic substrate. Details of the deconvoluted XPS signals for each modified surface are compiled in Table S5.

The N 1s well-resolved spectra (Figure 3c,g,k) exhibited two distinct peaks in the 399.7–400.2 eV region for all modified substrates. These peaks correspond to C–N bonds, confirming the covalent anchoring of ADM molecules. The spectral pattern indicates that one amine terminus of each ADM linker forms a C–N bond with the GC substrate, whereas the other interacts with the ERGO layer, demonstrating the dual-binding nature of the diamine molecules [15,16]. The absence of additional peaks attributable to free or protonated amines confirms that both amino groups are chemically bound, resulting in the formation of robust and stable SAMs.

On the ERGO-functionalized surfaces, the O1s spectrum revealed multiple components associated with oxygen-containing functionality (Figure 3d,h,l). C–O bonds in hydroxyl and epoxy moieties were assigned peaks between 530.9 and 531.1 eV, while those around 531.5 and 531.7 eV correspond to carbonyl (C=O) groups. There are signals between 532.2 and 533.4 eV attributed to carboxyl species (O-C=O), and a peak in binding energy at approximately 535.6–535.7 eV attributed to molecular oxygen (H_2_O/O_2_). Finally, the XPS results demonstrate that SAMs derived from ADMs can be anchored on GC substrates [3,15]. Additionally, GO can be electrochemically reduced to ERGO. The coexistence of distinct C-N and C-O bonding states leads to a chemically stable, covalently interconnected surface structure ideal for durable and efficient electrochemical sensing.

### 3.5. Morphological Analysis of AFM and SEM

**Figure 4:**
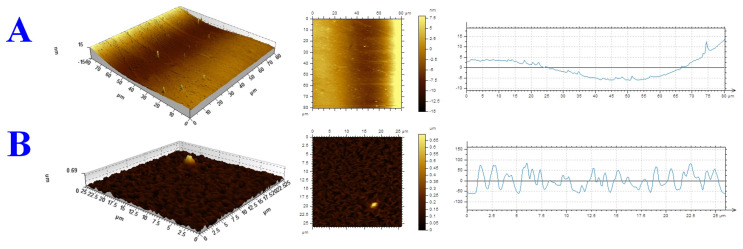
Three-dimensional AFM topographs recorded in tapping mode with height profiles for (**A**) the pristine GC surface and (**B**) the p-PDA-modified GC surface.

AFM was used to examine the surface morphology of the GC substrate before and after modifying it with a SAM of p-PDA functionalization. The pristine GC surface was relatively smooth, with a root-mean-square roughness (R_p_) around 20.4 nm (Figure 4A). After the p-PDA layer was formed on the GC (Figure 4B), the surface became much rougher, and R_p_ increased to approximately 97.0 nm. It is clear from this dramatic increase in roughness that the p-PDA molecules hook onto the surface of the GC, creating a highly textured coating. To interpret the morphological transformation at the molecular scale, the size of the p-PDA molecules was considered. Each carbon–carbon bond in a benzene ring is about 1.34 Å in length. With para-substitution, p-PDA conjugated molecular backbone spans roughly 6.7 Å from end to end. It is known that AFM tends to overestimate film thickness due to factors like molecular aggregation, probe tip convolution, and other instrumental limitations. Taking these factors into account, the observed roughness increase of about 77 nm (the difference between the modified and bare GC surfaces) is consistent with the addition of a continuous, ordered monolayer of p-PDA. This strong correlation between the experimental AFM data and the theoretical molecular dimensions suggests that a uniform and well-organized p-PDA monolayer has been established. It is important to maintain interfacial stability and to achieve superior electrochemical sensing characteristics through such a molecular assembly [15,54,55].

**Figure 5:**
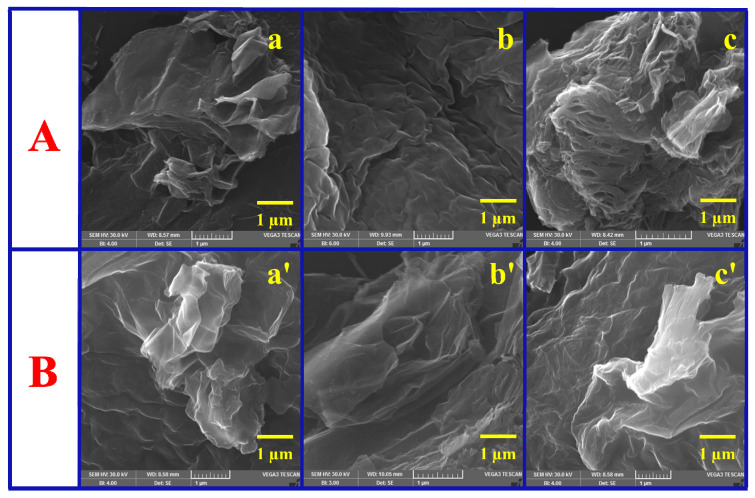
SEM micrographs illustrating the surface morphology (**A**) before and (**B**) after ERGO deposition on (**a**,**a’**) o-PDA-, (**b**,**b’**) m-PDA-, and (**c**,**c’**) p-PDA-functionalized GC surfaces.

SEM imaging was used to look at how the shapes of GO and ERGO surfaces changed on GC substrates that had different ADM linkers (o-PDA, m-PDA, and p-PDA) attached to them. The SEM images (Figure 5A) show the usual wrinkled and crumpled look of exfoliated GO sheets that are spread out randomly across the GC surface in GO-modified substrates (GC/o-PDA/GO, GC/m-PDA/GO, and GC/p-PDA/GO). The sheets often have a curled texture, which suggests that some GO nanosheets have partially restacked due to van der Waals interactions [3,27]. An electrochemical reduction of the GO surface to ERGO caused changes in its shape (Figure 5B). The GC/ADM/ERGO surfaces now have a texture that is more folded and wavier, which is what happens when ERGO is reduced. This corrugated shape is very similar to the one that can be made with traditional chemical or thermal reduction methods. The ERGO layers that are connected by the ADM linkers look smoother and more even than the original GO layers.

This suggests that the film became denser and the interface became more even after it was reduced. The fact that the diamine linkers used to hold the GO together are aromatic is what made the surface smoother and more even. These linkers have benzene-ring backbones that allow for strong π–π stacking interactions with the graphene sheets. This probably makes the space between the layers bigger and the surface defects smaller. This conjugated linker structure makes the process of turning GO into ERGO more orderly. Also, better π–π coupling between graphene sheets that are next to each other during electrochemical reduction may cause the sheets to stick together a little bit, which would make the film structure more coherent with fewer defects [15,31,56]. The combined AFM and SEM results show that the ADM linkers were successfully added to the GC surface and that GO was effectively turned into a layer of ERGO on the GC substrates. The film morphology is dense, even, and strong, which is perfect for making sure the interface stays stable and is very useful for future electrochemical sensing applications.

### 3.6. Electrochemical Characterization of ADM-Modified Electrodes Before and After ERGO Attachment

The electrochemical behavior of SAMs formed by the three ADM isomers (o-PDA, m-PDA, and p-PDA) on GC-electrodes was examined by CV. The measurements were performed using 1 mM Ru(NH_3_)_6_Cl_3_ in 0.2 M PBS (pH 3) at a scan rate of 50 mV s^−1^. As shown in Figure S3A, an unmodified bare GC-electrode yields a well-defined and reversible [Ru(NH_3_)_6_]^3+/2+^ redox couple with a peak-to-peak separation (ΔE_p_) of approximately 69 mV, which is typical of a diffusion-controlled electron transfer process. Upon modifying the GC electrode with an ADM monolayer, the ΔE_p_ increased in the order: bare GC (69 mV) < GC/o-PDA (71 mV) < GC/m-PDA (73 mV) < GC/p-PDA (87 mV) electrodes (Table S6). This rise in ΔE_p_ was accompanied by a concomitant decrease in the redox peak current. These changes suggest a rise in interfacial resistance, probably due to electrostatic repulsion between the positively charged [Ru(NH_3_)_6_]^3+/2+^ probe and the protonated surface amine groups (–NH_3_^+^) that appear when diamines undergo a Michael-type nucleophilic addition to the carbon surface [26].

The positional isomerism of the ADM molecules strongly affects the packing and order of the resulting SAM. The para isomer (p-PDA) has a symmetric molecular configuration that promotes the formation of a densely packed, highly ordered monolayer with minimal steric hindrance. Consequently, the p-PDA produced the largest ΔE_p_ and the lowest current response among the isomers. In contrast, the o-PDA and m-PDA isomers (with more non-linear structures) form less ordered monolayers, which are more permeable to the redox species [15,32,57,58]. As a result, the blocking effectiveness of the SAMs followed the trend p-PDA > m-PDA > o-PDA. Electron-transfer kinetics are influenced by a combination of molecular geometry, monolayer packing density, and interfacial electrostatic forces [57,58].

The CV with the same redox probe (Figure S3B) showed that GO was successfully immobilized on the ADM-functionalized GC electrodes. After GO was deposited, both the reduction peak current and the ΔE_p_ showed small increases, which means that the way electrons were transferred changed (and became a little harder). The ΔE_p_ values for the GO-modified electrodes were GC/o-PDA/GO (70 mV), GC/m-PDA/GO (77 mV), and GC/p-PDA/GO (78 mV). This increase in ΔE_p_ is in line with GO’s naturally low electrical conductivity, which makes electrons harder to transfer. Assembling GO is also affected by the SAM architecture. The relatively higher ΔE_p_ for GC/p-PDA/GO electrode can be attributed to the tightly packed p-PDA monolayer, further restricting access of the redox probe to the electrode surface [15].

Electrochemical reduction of GO to ERGO improved electron transfer on all ADM-modified electrodes (Figure S3C). The CV responses of the GC/o-PDA/ERGO, GC/m-PDA/ERGO, and GC/p-PDA/ERGO electrodes all showed much higher currents, which indicated that the ERGO network had restored conductive pathways. This result backs up earlier research that showed that conductive nanomaterials, like graphene derivatives, can restore faradic activity at SAM interfaces that were previously insulated. The ERGO layer is probably an electron mediator in the ADM framework. This makes it easier for charges to transfer between the electrode and the electrolyte [3,34]. The Randles–Ševčík equation [59] was employed to find the electroactive surface area (EASA). For the bare GC electrode, it was 0.063 cm^2^. For the GC/o-PDA/ERGO, GC/m-PDA/ERGO, and GC/p-PDA/ERGO electrodes, it went up to 0.065, 0.068, and 0.087 cm^2^, respectively (Table S6). Because of the densely packed SAM structure and the high conductivity of ERGO, the p-PDA-based system has a much higher EASA than its o-PDA and m-PDA counterparts [57,58].

### 3.7. Analysis of ET Dynamics Using EIS

**Figure 6:**
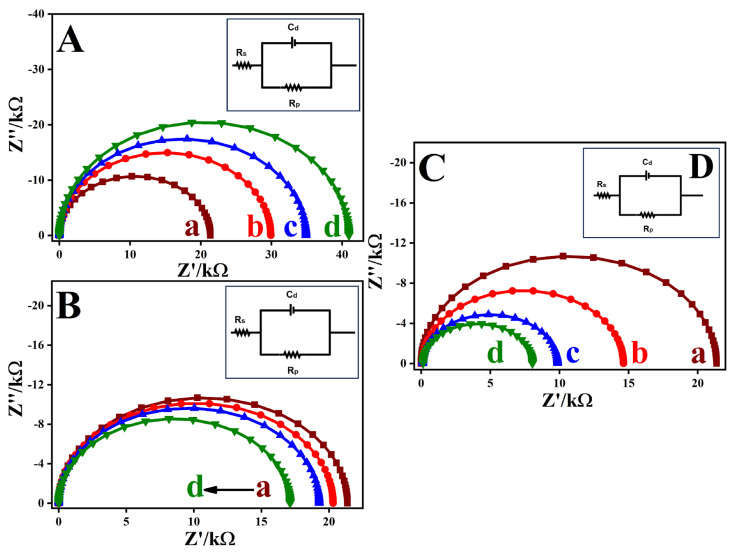
Nyquist plots obtained for (**A**) (a) bare GC, (b) GC/o-PDA, (c) GC/m-PDA, and (d) GC/p-PDA; (**B**) GO- and (**C**) ERGO-modified electrodes formed on (a) bare GC, (b) o-PDA-, (c) m-PDA-, and (d) p-PDA-functionalized surfaces. Measurements were performed in a 1 mM [Ru(NH_3_)_6_]^3+^/^2+^ redox system containing 0.2 M PBS (pH 3) within the 0.01 Hz–100 kHz frequency range. Inset (**D**): Equivalent circuit representations used for data fitting.

EIS was employed to investigate the electron transfer (ET) behavior of GC-electrodes modified with ADM isomers of different structural configurations. Figure 6A shows the Nyquist plots for bare GC, GC/o-PDA, GC/m-PDA, and GC/p-PDA electrodes recorded in 0.2 M PBS (pH 3) containing 1 mM [Ru(NH_3_)_6_]Cl_3_ over the frequency range 0.01 Hz–100 kHz. The impedance data were fitted using SAI ZView 3.20 software (Scribner Associates, Inc., Southern Pines, NC, USA) based on a modified Randles equivalent circuit, R_s_(C_dl_–R_p_), shown in the inset of Figure 6A, where R_s_ represents the solution resistance, C_dl_ the double-layer capacitance, R_p_ the parallel resistance, and R_ct_ the charge-transfer resistance. The electronic parameters derived from impedance measurements are summarized in Table S7. The Nyquist plots exhibited semicircular regions in the high-frequency domain corresponding to charge-transfer processes at the electrode–electrolyte interface. The semicircle diameter reflects R_ct_, which serves as an indicator of ET kinetics. Among all samples, GC/p-PDA electrode exhibited the largest semicircle, corresponding to the highest R_ct_ (42.8 kΩ), consistent with the formation of a densely packed SAM that restricts electron flow. As observed in R_ct_, GC/p-PDA > GC/m-PDA > o-PDA > bare GC followed the order of electrodes, proving that molecular ordering and compactness within the SAM control interfacial charge transport [15,57,58].

The increase in Rct after ADM modification confirms the successful formation of insulating organic monolayers that impede access of the redox probe to the electrode surface. In a highly ordered monolayer, p-PDA combines well with sp^2^ domains of carbon, forming a tightly packed and highly ordered structure. While m-PDA and o-PDA structural isomeric geometry introduces steric constraints, resulting in low packing efficiency and coverage. It is evident from these observations that molecular geometry plays an important role in determining SAM density and ET resistance. The heterogeneous ET rate constants (ket) were determined for each system [59], yielding values of 1.78 × 10^−3^, 1.27 × 10^−3^, 1.09 × 10^−3^, and 9.3 × 10^−4^ cm s^−1^ for bare GC, GC/o-PDA, GC/m-PDA, and GC/p-PDA electrodes, respectively (Table S7). The progressive decline in ket from the bare electrode to the para-substituted system confirms that increasing SAM compactness suppresses direct ET between the redox probe and the electrode surface [57,58].

To study how electrons transfer at the interface, GO surfaces were attached to GC electrodes that were treated with o-PDA, m-PDA, and p-PDA, as shown in Figure 6B. The EIS showed that the heterogeneous electron-transfer rate constants (k_et_) for the [Ru(NH_3_)_6_]^3+^/^2+^ couple increased slightly from 1.78 × 10^−3^ cm s^−1^ on bare GC to 1.88 × 10^−3^, 1.98 × 10^−3^, and 2.22 × 10^−3^ cm s^−1^ on GC/o-PDA/GO, GC/m-PDA/GO, and GC/p-PDA/GO electrodes, respectively (Table S7). Although GO is generally viewed as a poor conductor, it contains residual sp^2^-hybridized domains that allow partial conductivity, and electrodes modified with GO can display charge-transfer resistance comparable to electrodes decorated with reduced ERGO and lower than bare GC. This partial conductivity and the covalent linkage of GO to the self-assembled diamine layers, therefore, improve electron-transfer kinetics [34,35,36,37].

Further enhancement was obtained by electrochemically reducing the GO functionalization to ERGO, as shown in Figure 6C. ADM-derived organic films form strong covalent bonds and provide efficient electron-transfer channels. Anchoring ERGO to such surfaces increases conductivity and introduces additional active sites for redox reactions. Consistent with this, Nyquist plots for GC/o-PDA/ERGO, GC/m-PDA/ERGO, and GC/p-PDA/ERGO electrodes showed much smaller semicircles than those for GO-modified electrodes, and the ket values rose to 2.62 × 10^−3^, 3.90 × 10^−3^, and 4.81 × 10^−3^ cm s^−1^, respectively (versus 1.78 × 10^−3^ cm s^−1^ on bare GC) (Table S7). The ERGO network evidently establishes conductive pathways through the monolayer, greatly facilitating charge transport.

Among the three ADM isomers, the GC/p-PDA/ERGO electrode exhibited the smallest charge-transfer resistance and the highest k_et_. The p-PDA adopts a linear configuration that promotes symmetric packing of the monolayer, uniform anchoring of the ERGO sheets, and strong π–π interactions between the aromatic linkers and the ERGO basal plane. In contrast, the bent structures of the m-PDA and o-PDA isomers disturb molecular order and diminish conductivity. Quantum-interference studies of benzene junctions show that changing the connection from para to meta suppresses conductance due to destructive interference. Likewise, comparative studies of single-molecule junctions report that meta-substituted benzene rings act as π-insulators and exhibit conductance several orders of magnitude lower than their para-substituted counterparts [32,57,58,60]. In contrast, the angular structures of the meta- and ortho-linkers hinder ordered packing and disrupt the formation of efficient electron pathways. The observed trend in the present work (R_ct_(o-PDA) > R_ct_(m-PDA) > R_ct_(p-PDA)), therefore, reflects the intrinsic influence of molecular geometry on electron transport. As a result, coupling a linear p-PDA monolayer with a conductive ERGO coating results in a highly conductive interface. The optimized molecular structure of the self-assembled layer and the porous, highly conductive ERGO network work synergistically to facilitate electron transport, which is essential for achieving high-performance electrochemical sensors [15].

### 3.8. Electrocatalytic Reduction of NBz on ERGO-Modified Electrodes

**Figure 7:**
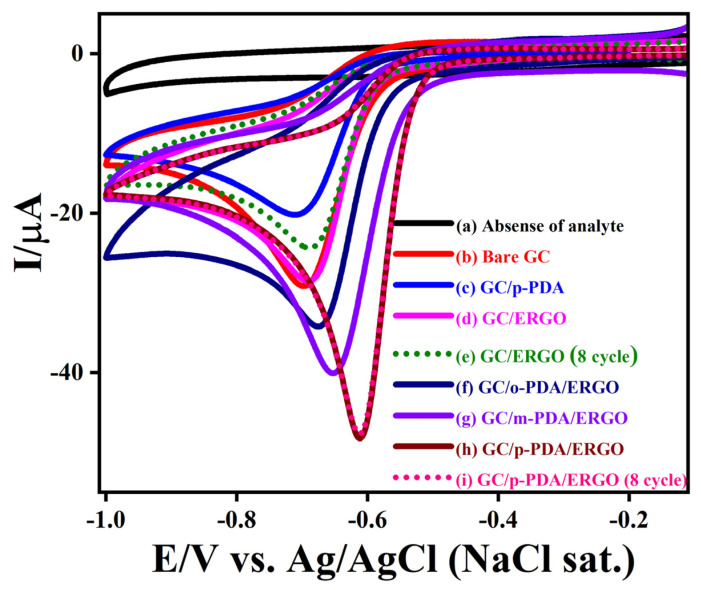
CVs recorded (a) in the absence and (b–i) in the presence of 0.5 mM NBz for (b) bare GC, (c) GC/p-PDA, (d) GC/ERGO, (e) GC/ERGO (8th cycle), (f) GC/o-PDA/ERGO, (g) GC/m-PDA/ERGO, (h) GC/p-PDA/ERGO, and (i) GC/p-PDA/ERGO (8th cycle). All measurements were performed in 0.2 M PBS (pH 7.2) at a scan rate of 50 mV s^−1^.

An assessment of NBz reduction activity on a series of GC electrodes modified with various assemblies, including GC/ERGO, GC/o-PDA/ERGO, GC/m-PDA/ERGO, and GC/p-PDA/ERGO, is shown in Figure 7. CV was conducted in 0.2 M PBS (pH 7.2) at a scan rate of 50 mV/s, and key results are summarized in Table S8. In blank electrolyte (no NBz analyte), all electrodes showed virtually no Faradaic current, indicating electrochemical stability in this range. Adding 0.5 mM NBz, the unmodified GC electrode exhibited a single cathodic peak at −0.69 V (−26.3 µA), corresponding to NBz reduction. The GC electrode modified only with p-PDA (which is essentially non-conductive) showed a reduction peak at −0.71 V but with a much lower current (−18.8 µA), reflecting the insulating character of p-PDA that hinders electron transfer. In comparison, the initiated electrode (GC/ERGO) displayed NBz reduction at −0.69 V with an initial peak current of −26.4 µA (curve d). However, the reduction current diminished to −22.5 µA upon continuous cycling, indicating this system has limited operational stability.

To boost electron-transfer kinetics and film durability, ERGO was anchored to the sheets on GC via ADM linkers. Both GC/o-PDA/ERGO and GC/m-PDA/ERGO electrodes showed enhanced catalytic behavior. Their NBz reduction peaks appeared at −0.67 V (−30.6 µA) and −0.65 V (−36.5 µA), respectively. Notably, the GC/p-PDA/ERGO electrode yielded a sharp cathodic peak at −0.61 V with a peak current of −46.5 µA. This substantial positive shift and current increase imply that the p-PDA layer promotes a more effective ERGO film assembly and faster electron transport during NBz reduction. Moreover, the GC/p-PDA/ERGO electrode maintained nearly identical NBz reduction peaks over eight successive CV scans, indicating excellent stability and reproducibility. In addition to its superior catalytic efficiency, p-PDA/ERGO exhibits a high current density, low negative reduction potential, and stable cycling characteristics.

This improvement arises from a larger electroactive surface area and better charge transport provided by the uniform, dense ERGO coverage on the well-ordered p-PDA layer. On the other hand, the GC/p-PDA modified electrode displayed a more negative reduction potential and a lower current (−18.7 A), indicating poor conductivity and poor electron transfer. Attaching ERGO on the p-PDA monolayer dramatically improved charge transport, reflecting a strong synergistic interaction between the conductive ERGO sheets and the structured compact layer. Likely, the reduced defect density and symmetric para-substitution of p-PDA facilitate tight π–π stacking and homogeneous ERGO integration, which enable rapid electron transfer and efficient NBz conversion. Under these neutral conditions, NBz is reduced via a four-electron pathway to phenylhydroxylamine (Scheme S1). All electrodes tested showed a significant reduction reaction with NBz, but the GC/p-PDA/ERGO electrode had the highest current and the least negative shift. The anchoring of ERGO on GC/p-PDA decreases the R_ct_ and increases interfacial conductivity, so that the performance of the device improves. NBz reduction intermediate and surface fouling are easily handled by GC/p-PDA/ERGO electrodes since the peak persists for several cycles. These results show that the GC/p-PDA/ERGO electrode is a very stable, strong, and effective NBz reduction system. This suggests that it could be used for long-lasting and high-performance electrocatalytic sensing applications.

**Figure 8:**
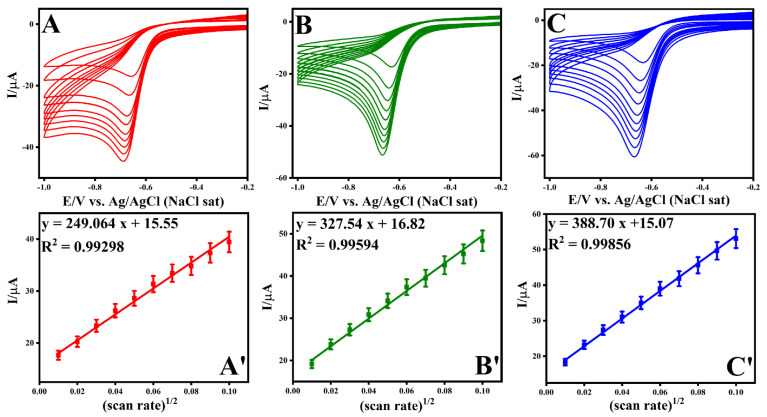
Evaluation of scan rates and the resulting plots of current versus the square root of the scan rate for various structural isomers of electrode configurations: (**A**,**A’**) GC/o-PDA/ERGO, (**B**,**B’**) GC/m-PDA/ERGO, and (**C**,**C’**) GC/p-PDA/ERGO electrodes.

Throughout a scan rate range of 0.01–0.1 Vs^−1^, Figure 8 displays the continual reduction of NBz at modified electrodes made of ADM-facilitated ERGO connected electrodes in a 0.2 M PBS (pH 7.2) containing 0.5 mM NBz. The correlation coefficients (R^2^) for o-PDA, m-PDA, and p-PDA-mediated ERGO attached electrodes were 0.99298, 0.99594, and 0.99856 (Figure 8), respectively, indicating the observed NBz reduction at all of these modified electrode types was a diffusion-controlled process. The peak current vs. the square root of scan rates plot for NBz reduction was linear [15,61,62,63,64,65,66,67,68,69,70,71].

### 3.9. Sensitive Analysis of NBz

**Figure 9:**
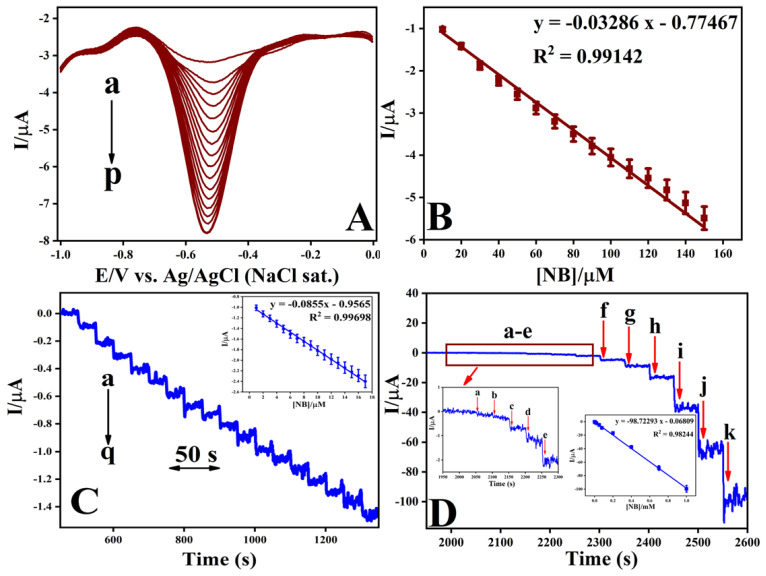
(**A**) DPV responses for NBz recorded at the GC/p-PDA/ERGO electrode in 0.2 M PBS (pH 7.2) with successive additions of NBz in 10 μM increments. (**B**) Corresponding calibration plot showing the relationship between peak current and NBz concentration. (**C**) Amperometric i–t response for NBz detection at the GC/p-PDA/ERGO electrode with successive additions of 1 μM NB (a–q). (**D**) Amperometric response recorded for successive additions of NBz at concentrations of 0.0005, 0.002, 0.006, 0.008, 0.02, 0.05, 0.08, 0.2, 0.4, 0.7, and 1 mM in 0.2 M PBS (pH 7.2).

A DPV experiment was carried out to evaluate the reduction of NBz on a GC-electrode modified with p-PDA/ERGO (Figure 9A). Measurements were made in 0.2 M PBS at pH 7.2. The 0.5 mM of NBz electrochemical reduction was detected as a distinct cathodic peak at roughly −0.57 V (curve b). Successive 10 μM increments of NBz up to 150 μM caused the cathodic peak current to rise linearly, while its potential remained essentially unchanged. This behavior indicates stable electron transfer kinetics and good interfacial conductivity at the modified surface. The calibration plot is shown in Figure 9B, revealing a strong linear association of peak current with NBz concentration, with a correlation coefficient of 0.99142. This illustrates the electrode’s consistency of results and sensitivity to quantitative detections. The electrode’s consistent peak potential reflects its operational stability and strong resistance to surface fouling during successive cycles. The p-PDA/ERGO composite exhibits remarkable electrocatalytic activity and mechanical durability. Owing to its high accuracy and responsiveness, this electrochemical approach serves as a reliable method for detecting nitroaromatic contaminants in environmental samples.

Electrochemical performance analysis via amperometric analysis (Figure 9C) demonstrates the current transients obtained using a GC/p-PDA/ERGO electrode immersed in aerated 0.2 M PBS at pH 7.4 under magnetic stirring conditions, monitored at a reduction potential of −0.57 V. Injection of NBz commencing at 0.001 mM triggered a rapid increase in faradaic current that achieved equilibrium within 3 s (Figure 9C, curve a). The electrochemical signal exhibited direct linearity with respect to analyte concentration throughout the 0.001–0.017 mM range, yielding a calibration coefficient R^2^ = 0.99698. Prolonged current–time measurements (Figure 9D) confirmed that the amperometric response of the GC/p-PDA/ERGO electrode remains linear over a much wider NBz concentration range of 0.5–1000 μM, with an improved correlation coefficient of 0.98244. The rapid return to a stable current following each NBz addition shows that electron transfer is efficient and surface fouling is negligible during repeated assays. Using the standard deviation method (3σ/S), the limit of detection was calculated to be 0.040 µM, and the electrode sensitivity was determined to be 1410 μA mM^−1^ cm^−2^. These figures represent a substantial improvement over both unmodified GC and ERGO electrodes. The amplified electroanalytical capability derives from the cooperative effect of the electron-conductive ERGO matrix coupled with the precisely organized p-PDA adlayer, which establishes a chemically stable substrate with enhanced accessibility that accelerates the electrochemical conversion of NBz molecules. As documented in Table 1, the GC/p-PDA/ERGO electrode system showcases exceptional analytical attributes, namely exceptionally diminished detection thresholds, an extensive working concentration domain, and sustained performance integrity across extended operational periods, making it a compelling electrochemical platform for the sensitive determination of NBz and analogous organic contaminants in environmental matrices.

Experimental observations reveal that ERGO forms a stable and strong interface with GC electrodes functionalized through self-assembly of p-PDA. It achieves a detection limit of 0.040 µM using the GC/p-PDA/ERGO electrode configuration for NBz sensing. The electrodes modified with ADA linkers and ERGO integration showed even lower sensitivity in our previous report [15]. The performance differences between ADA- and ADM-based systems can be attributed to steric effects, molecular flexibility, and packing efficiency of the SAMs, which strongly influence nanomaterial immobilization and electron-transfer kinetics [72,73,74]. ADMs tend to possess relatively rigid aromatic backbones, which can influence their interaction with GC surfaces by limiting conformational flexibility during self-assembly. In the case of PDA isomers (o-PDA, m-PDA, and p-PDA), the rigid benzene ring framework can introduce steric constraints that affect molecular packing density, orientation of amine functionalities, and accessibility for subsequent nanomaterial immobilization, rather than preventing surface binding outright.

### 3.10. Selective Detection of NBz Using Amperometry

The GC/p-PDA/ERGO electrode was examined for selectivity toward NBz by monitoring its response in the presence of common inorganic and organic interferents (Figure S4). Initially, the addition of 3 μM NBz resulted in a sharp and immediate increase in reduction current, confirming the sensor’s sensitivity. To evaluate the effect of competing ions, the electrolyte was spiked with 30 μM each of typical cations (Na^+^, K^+^, Cu^2+^, Mg^2+^, and Ca^2+^) and anions (NO_3_^−^, Cl^−^, F^−^, SO_4_^2−^, and C_2_O_4_^2−^). A subsequent 3 μM NBz addition produced another clear current increase, indicating that these ions had a negligible impact on the signal. To assess selectivity, 3 μM concentrations of different organic molecules, aniline, p-nitrophenol, p-cresol, benzonitrile, and 4-nitrotoluene were introduced. These compounds produced only slight variations in current response, indicating negligible interference. The findings confirm that the GC/p-PDA/ERGO sensor exhibits outstanding specificity toward NBz detection, retaining consistent accuracy even within chemically diverse matrices. The GC/p-PDA/ERGO is highly dependable for monitoring NBz in real-world settings due to its high tolerance to both ionic and organic interferents.

### 3.11. Practical Applications

Translating electrochemical sensors from the laboratory to practical settings requires devices that are repeatable, reproducible, and stable. To evaluate these attributes, DPV was performed on GC/p-PDA/ERGO electrode, all prepared under identical conditions for NBz detection. Figure S5 summarizes the repeatability, reproducibility, and stability of the modified electrodes to NBz. For the repeatability assessment (Figure S5A), sequential DPV measurements were carried out on 10 μM NBz in 0.2 M PBS at pH 7.2. Over six successive runs, the cathodic peak consistently appeared near −0.57 V, with a relative standard deviation (RSD) of 1.27%, demonstrating excellent repeatability. Reproducibility tests using six independently fabricated GC/p-PDA/ERGO electrodes produced an RSD of 1.41%, indicating high fabrication consistency and analytical precision across multiple devices (Figure S5B). The stability was also evaluated using the modified sensor for 25 days, and its current response was tested for a five-day period in 10 μM NBz (Figure S5C). After 25 days, the electrode response was 96.1% of its starting value, suggesting that the GC/p-PDA/ERGO sensor possesses long-term stability.

**Figure 10:**
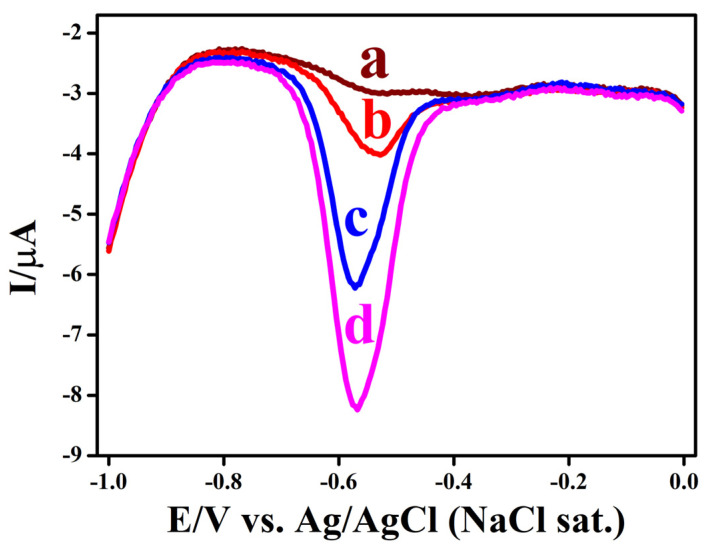
DPVs show the (a) river water, and after the addition of (b) 10 µM, (c) 80 µM, and (d) 150 µM NBz, measured at the GC/p-PDA/ERGO in a 0.2 M PB solution with a pH of 7.2.

The practical performance of the sensor was tested by analyzing NBz in river water using a standard addition approach (Figure 10). In the river sample, no reduction peak appeared (curve a), indicating that NBz was absent in the collected water. Following fortification of the sample matrix with 10, 80, and 150 μM NBz (curves b–d), well-defined cathodic peaks of incrementally increasing magnitude were recorded, confirming quantitative extraction efficiency. The corresponding analyte recovery values, as presented in Table S9, reached 99.0%, 99.8%, and 99.9%, accompanied by inter-measurement relative standard deviations of 1.01%, 0.071%, and 0.038%, respectively. This experimental evidence validates the exceptional analytical accuracy, measurement consistency, and operational dependability of the GC/p-PDA/ERGO electrode platform. As a result of its near-stoichiometric recovery rates and minimal inter-replicate variance, this method is well-suited to quantitatively quantify trace NBz in water matrices. As a result of the electrode’s superior performance stability, excellent signal reproducibility, and superior measurement fidelity, it has sustained performance stability. Therefore, it can be directly applied to authentic samples and continuous environmental monitoring.

## 4. Conclusions

In this investigation, SAMs of ADMs were fabricated on GC electrodes, and their structural and electrochemical properties were thoroughly characterized. Evidence from CV, EIS, and ATR-FTIR techniques confirmed that the different structural isomers of these diamine linkers were covalently attached to the electrode surface stably and uniformly. Evaluation using the [Ru(NH_3_)_6_]^3+/2+^ redox couple showed that the particular isomer of the diamine has a decisive impact on electron transfer behavior. Of the three isomers studied, the SAM formed from p-PDA via a Michael-type nucleophilic addition produced the most compact and well-ordered interface, as further demonstrated by AFM and Raman spectroscopy. Subsequent integration with ERGO was attained efficiently, as confirmed by various analytical methods. The p-PDA monolayer provided an excellent template for ERGO immobilization, resulting in a large electroactive surface, low R_ct_, and enhanced interfacial conductivity. As a result, the GC/p-PDA/ERGO electrode showed outstanding electrocatalytic activity for NBz reduction, reaching a low detection limit of 0.040 μM with excellent precision, stability, selectivity, and real-time applicability. These findings establish that molecular symmetry and substituent arrangement in diamine linkers directly control SAM ordering, nanomaterial assembly, and sensor performance. The mechanistic understanding gained here explains isomer-dependent behavior and offers a general framework for designing robust and high-conductivity electrochemical materials for environmental applications. Therefore, the GC/p-PDA/ERGO electrode assembly has been demonstrated to be a scalable and reliable framework for the development of next-generation sensing technologies and functional surface engineering.

## Figures and Tables

**Table 1:** Comparison of the electrochemical performance of NBz at GC/p-PDA/ERGO electrode with the previously reported electrodes.

Electrode	pH (PBS)	Linear Range (μM)	LOD (μM)	Reference
VSe2 ^a^/GCE ^b^	7	0.1–4	0.03	[61]
NHCPs-750 ^c^–GCE	7	5–2610	2.29	[62]
CMF-RGO ^d^/GCE	7	0.2–927.7	0.088	[63]
Ag-CuSe ^e^/GCE	7	0.1 to 400	0.01	[64]
GC/CA-AuNS ^f^	7	0.1–1000	0.0282	[65]
ZSO-gCN ^g^/GCE	7.2	30–900	2.2	[66]
Ni/Mo2C ^h^–GCE	7	2.11–1053.61	0.64	[67]
ZnO@c-GO ^i^ (2:3)/GCE	7.02	10–600	1.68	[68]
Co-NC-800 ^j^-GCE	7	0.1–6363–863	0.086	[69]
CuS-BCN ^k^/GCE	7	0.5–150, 150–1000	0.120	[70]
GO-AgNPs ^l^/GCE	7	0.5 to 900	0.261	[71]
GC/p-PDA/ERGO	7.2	0.5 to 1000	0.040	This work

^a^ Vanadium diselenide. ^b^ Glassy Carbon Electrode. ^c^ Nitrogen-doped hollow carbon nanospheres. ^d^ Reduced graphene oxide supported cellulose microfiber. ^e^ Silver-Doped Copper Selenide Composite. ^f^ Citric acid-capped gold nanostructures. ^g^ Zinc stannate incorporated graphitic carbon nitride nanocomposite. ^h^ Ni-doped coral-like three-dimensional Mo2C nanorod. ^i^ Zinc oxide-carboxylated graphene oxide composite. ^j^ Cobalt-embedded nitrogen-doped ordered macroporous carbon. ^k^ Flower-like copper sulfide-decorated boron-nitrogen co-doped carbon-modified. ^l^ Green synthesized silver nanoparticles decorated on reduced graphene oxide.

## Data Availability

The data that support the findings of this study are available from the corresponding author upon reasonable request.

## Supplementary Materials

The following supporting information can be downloaded at: https://www.mdpi.com/article/10.3390/bios16010033/s1, Figure S1: UV-vis spectrum obtained for GO; Figure S2: CVs illustrating the electrochemical reduction of GO on different electrodes in PBS; Figure S3: CVs recorded for bare and modified GC-electrodes in 0.2 M PBS (pH 3) containing 1 mM [Ru(NH3)6]^3+/2+^; Figure S4: selectivity analysis of NBz at GC/p-PDA/ERGO with various interfering compounds; Figure S5: Repeatability, reproducibility, and stability of GC/p-PDA/ERGO electrode for NBz sensing; Scheme S1: the possible reaction of the electrochemical reduction of NBz; Table S1: voltammetry responses obtained for the electrochemically reduced GO modified electrode at ADMs ERGO electrodes; Table S2: ATR-FT-IR spectra for GC/o-PDA, GC/m-PDA, and GC/p-PDA substrates and their assignments; Table S3: ATR-FT-IR spectra for GC/o-PDA/GO, GC/m-PDA/GO, and GC/p-PDA/GO and their assignments; Table S4: ATR-FT-IR spectra for GC/o-PDA/ERGO, GC/m-PDA/ERGO, and GC/p-PDA/ERGO and their assignments; Table S5: XPS spectra for GC/o-PDA/ERGO, GC/m-PDA/ERGO, and GC/p-PDA/ERGO and their assignments; Table S6: voltammetry responses for modified electrodes; Table S7: charge transfer resistance and electron transfer rate constant data of modified electrodes; Table S8: voltammetry responses obtained for electrocatalytic reduction of NBz at ArDAs ERGO electrodes; Table S9: determination of NBz in river water sample using GC/p-PDA/ERGO.
