# Supplementary material for: Potentiodynamic Fabrication of Aromatic Diamine Linkers on Electrochemically Reduced Graphene Oxide Surface for Environmental Pollutant Nitrobenzene Monitoring
Source: Biosensors (Basel). 2026 Jan 1;16(1):33. doi: 10.3390/bios16010033 (PMC12839025; doi:10.3390/bios16010033)
Supplement: Supplementary file 1 [file biosensors-16-00033-s001.zip › biosensors-4040079-supplementary.pdf]

Potentiodynamic Fabrication of Aromatic Diamine Linkers on Electrochemically Reduced Graphene Oxide Surface for Environmental Pollutant Nitrobenzene Monitoring

Karmegam Muthukrishnan[1,2], Venkatachalam Vinothkumar[3,4], Mathur Gopalakrishnan Sethuraman[2,*], and Tae Hyun Kim[4,*]

[1]Centre for Smart Energy Systems, Chennai Institute of Technology, Chennai, 600069, India

[2]Department of Chemistry, The Gandhigram Rural Institute-Deemed to be University, Gandhigram 624302, India

[3]Korea Native Animal Resources Utilization Convergence Research Institute, Soonchunhyang University, Asan 31538, Republic of Korea

[4]Department of Chemistry, Soonchunhyang University, Asan 31538, Republic of Korea

Authors:

Karmegam Muthukrishnan (E-mail: muthukrishnank.chem@citchennai.net)

Venkatachalam Vinothkumar (E-mail: vinothvr66@gmail.com)

***Corresponding Authors:**

Prof. Mathur Gopalakrishnan Sethuraman (E-mail: mgsethu@gmail.com)

Prof. Tae Hyun Kim (E-mail: thkim@sch.ac.kr)

No. of pages: 16, No. of figures: 5, No. of schemes: 1, No. of tables: 9

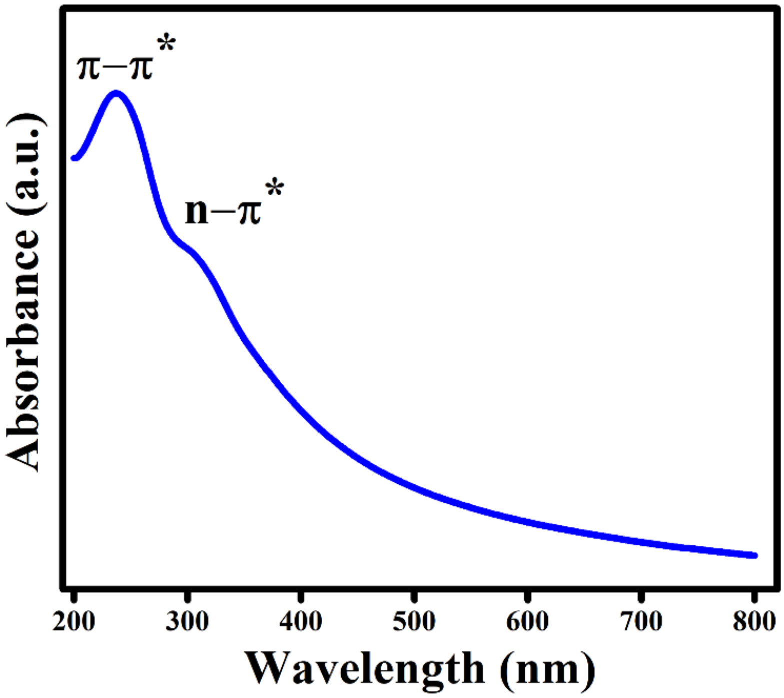

Fig. S1. UV-vis spectrum obtained for GO.

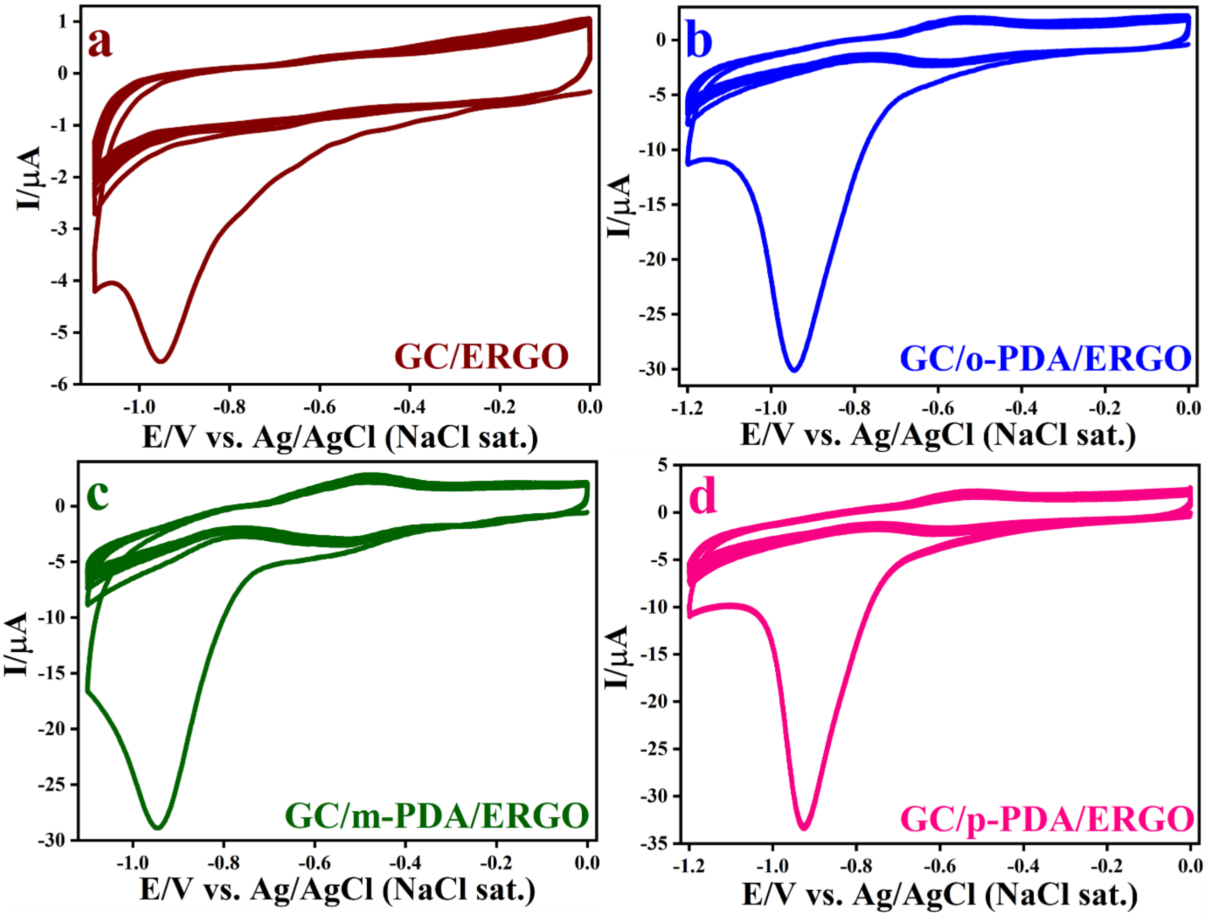

Fig. S2. CVs illustrating the electrochemical reduction of GO on (a) GC/ERGO, (b) GC/o-PDA/ERGO, (c) GC/m-PDA/ERGO, and (d) GC/p-PDA/ERGO electrodes. Measurements were carried out for 15 consecutive cycles in 0.2 M PBS (pH 7.2) at a scan rate of 100 mV s^{-1}.

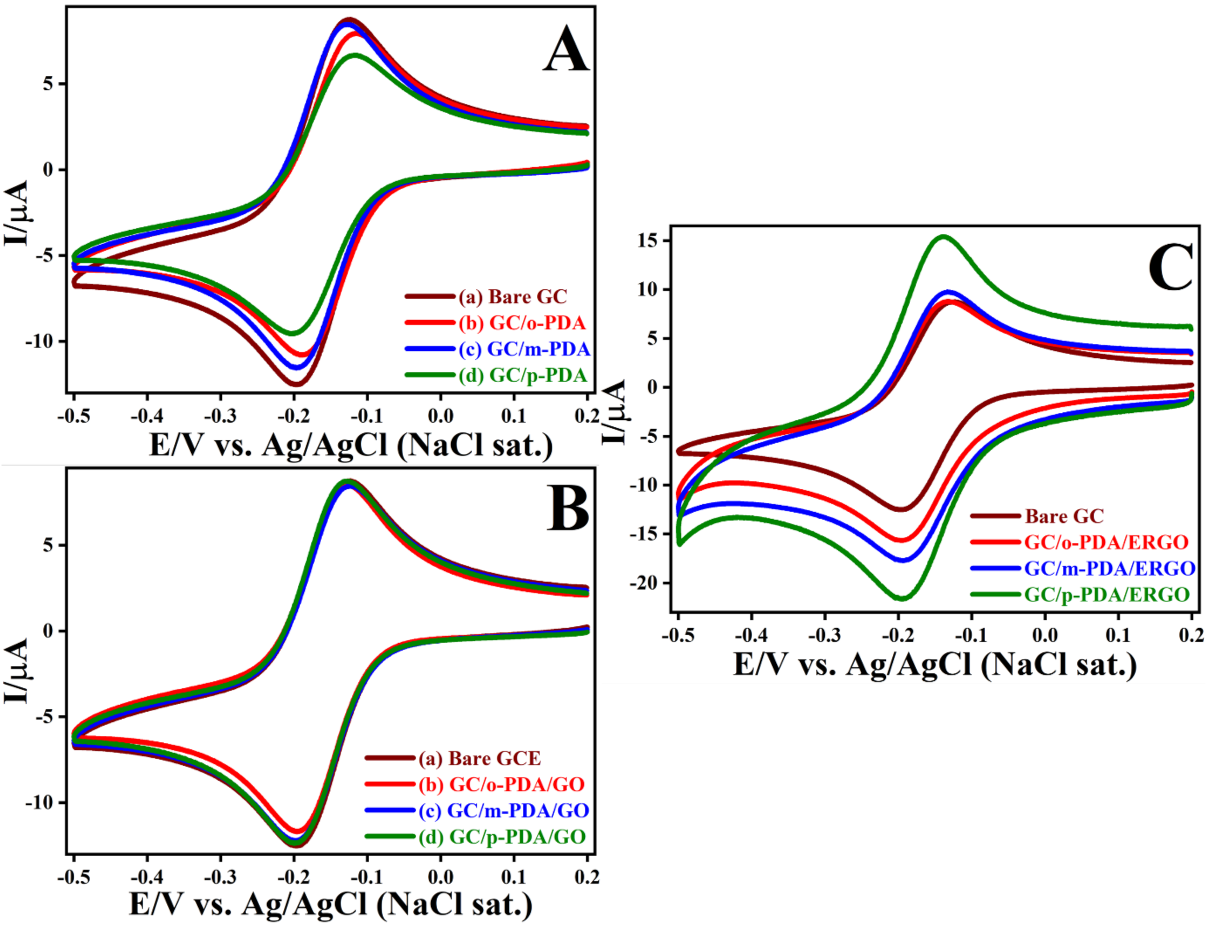

Fig. S3. CVs recorded for bare and modified GC-electrodes in 0.2 M PBS (pH 3) containing 1 mM $[Ru(NH_3)_6]^{3+/2+}$ at a scan rate of 50 mV/s. (A) CVs before GO attachment; (B) after GO immobilization; and (C) following electrochemical reduction of GO on ArDA-modified electrodes: (b) GC/o-PDA, (c) GC/m-PDA, and (d) GC/p-PDA.

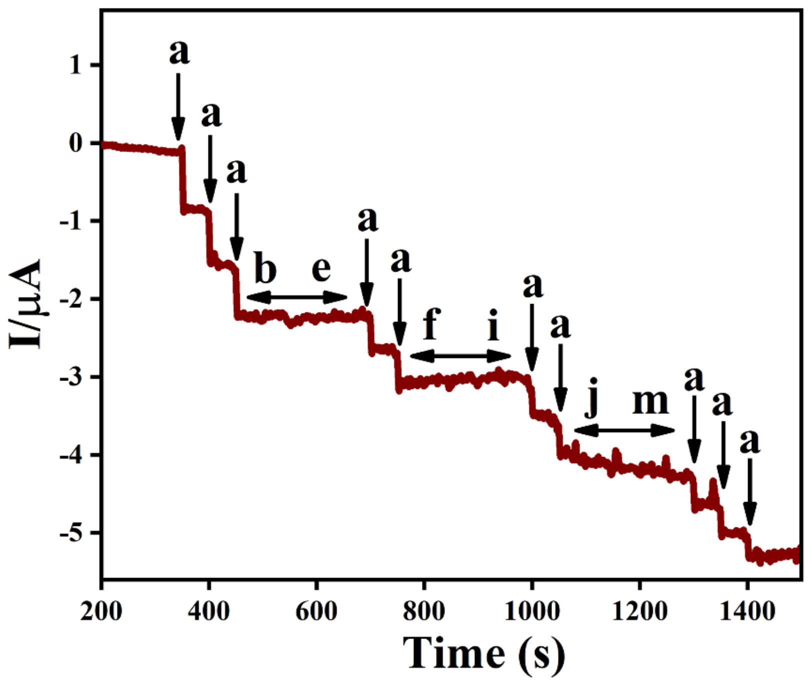

Fig. S4. Amperometric curve responses for the successive additions of (a) 3 μM NBz, (b-f) 30 μM each Na^{+}, K^{+}, Cu^{2+}, Cl^{-}, NO^{3+} (g-k) Mg^{2+}, Ca^{+2}, F^{-}, SO_4^{2-}, $C_2O_4^{2-}$, and 3 μM of major interferences like aniline, p-nitrophenol, p-cresol, benzonitrile, and 4-nitrotoluene at GC/p-PDA/ERGO electrode in 0.2 M PBS (pH 7.2) at an applied potential of -0.57 V.

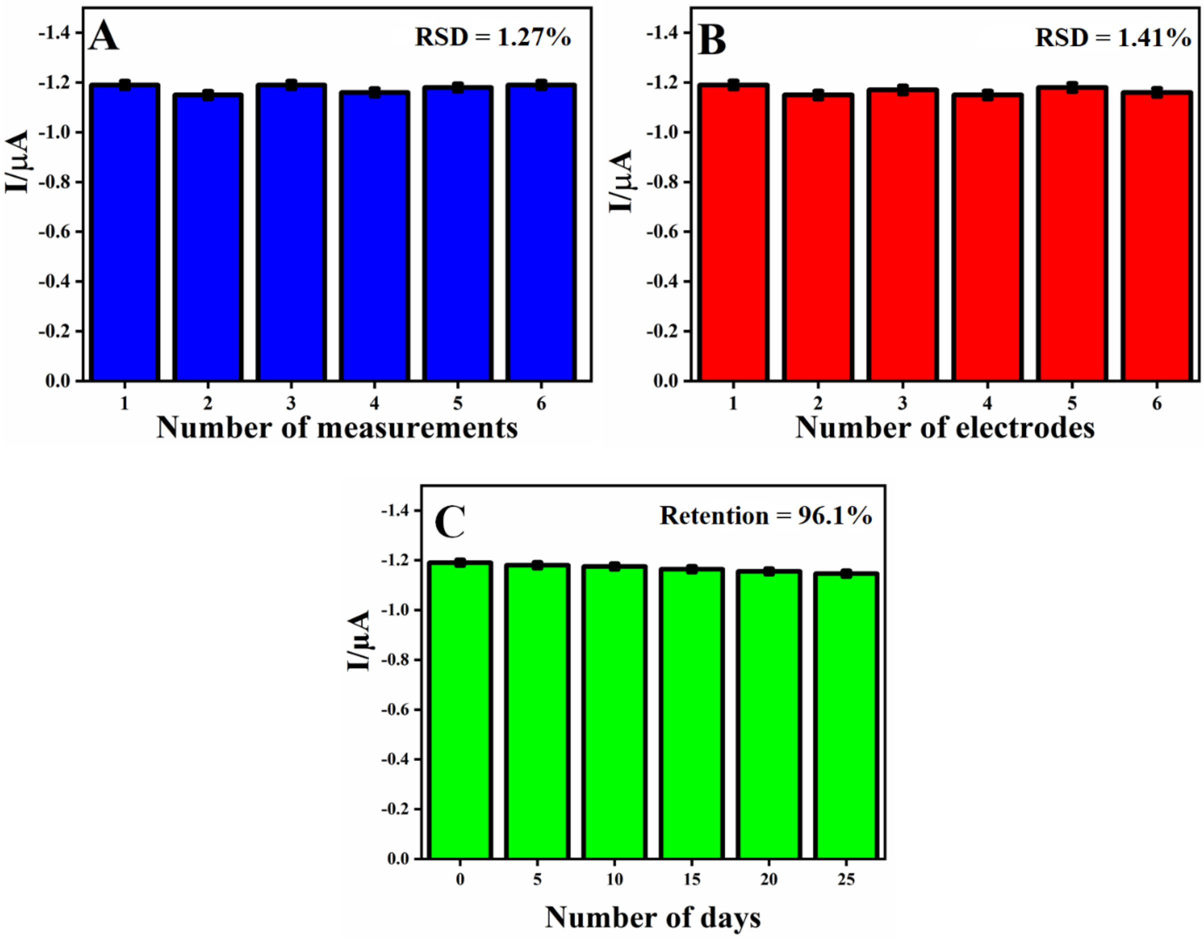

Fig. S5. Repeatability, reproducibility, and stability of GC/p-PDA/ERGO electrode for NBz sensing.

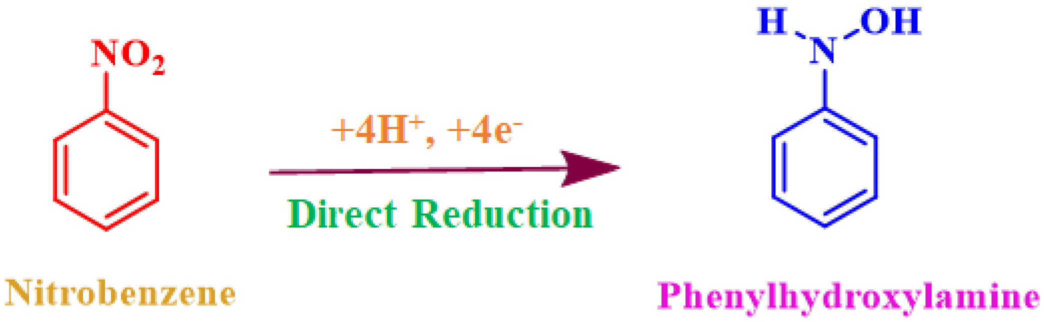

Scheme S1. The possible reaction of the electrochemical reduction of NBz.

Table S1. Voltammetry responses obtained for the electrochemically reduced GO modified electrode at ADMs ERGO electrodes.

Electrodes	Potential (mV)	Reduction current (µA)
**GCE/ERGO**	-0.953	-2
**GC/o-PDA/ERGO**	-0.943	-18
**GC/m-PDA/ERGO**	-0.945	-20
**GC/p-PDA/ERGO**	-0.925	-23

Table S2. ATR-FT-IR spectra for GC/o-PDA, GC/m-PDA, and GC/p-PDA substrates and their assignments.

ATR-FT-IR spectra absorption bands			Assignment
**GC/o-PDA (cm^{-1})**	**GC/m-PDA (cm^{-1})**	**GC/p-PDA (cm^{-1})**	
3213-3385	3254-3395	3287-3375	-N-H Stretching of ArDAs

2963-3033	2900-3033	2953-3013	C-H stretching of the aromatic ring
1626	1610	1626	NH_2 bending
1595	1495	1505	Aromatic C=C
1154-1424	1154-1314	1124-1314	C-N stretching
752-770	780–810	830–860	1,2 – Ortho<br>1,3 – Meta<br>1,4 – Para positions

Table S3. ATR-FT-IR spectra for GC/o-PDA/GO, GC/m-PDA/GO, and GC/p-PDA/GO and their assignments.

**ATR-FT-IR spectra absorption bands**			**Assignment**
**GC/o-PDA/GO (cm^{-1})**	**GC/m-PDA/GO (cm^{-1})**	**GC/m-PDA/GO (cm^{-1})**	

3105-3456	3175-3490	3155-3436	O–H stretching + N–H stretching
2900-2924	2893-2900	2895-2944	C-H stretching of the aromatic ring
1707	1700	1714	C=O stretching (carboxyl)
1626	1616	1606	N–H bending (scissoring)
1546	1546	1506	C=C aromatic ring stretching
1405-1250	1405-1250	1405-1250	C-N bond from ArDAs
1050–1250	1040-1240	1050-1250	C–O stretching (epoxy/alkoxy)
711-920	772-930	711-943	Diagnostic of ArDA substitution pattern (1,2-,1,3-,1,4-)

Table S4. ATR-FT-IR spectra for GC/o-PDA/ERGO, GC/m-PDA/ERGO, and GC/p-PDA/ERGO and their assignments.

**ATR-FT-IR spectra absorption bands**			**Assignment**
**GC/o-PDA/ERGO (cm^{-1})**	**GC/m-PDA/ERGO (cm^{-1})**	**GC/m-PDA/ERGO (cm^{-1})**	
3181-3365	3163-3342	3125-3345	Shifting of -N-H stretching secondary amine attached to the carbon surface
2908-2985	2864-2947	2856-2938	C-H stretching of the aromatic ring
1689	1683	1702	N–H bending (scissoring)
1562	1546	1556	Aromatic C=C
1496-1200	1496-1210	1488-1200	C–N bond from ArDAs

1340-1133	1351-1162	1326-1132	C-N stretching of ArDAs
720-950	705-929	705-925	Retained; indicates ArDA substitution pattern (1,2-,1,3-,1,4-)

Table S5. XPS spectra for GC/o-PDA/ERGO, GC/m-PDA/ERGO, and GC/p-PDA/ERGO and their assignments.

**Binding energy values**				**Assignment**
**Components**	**GC/o-PDA/ERGO (eV)**	**GC/m-PDA/ERGO (eV)**	**GC/p-PDA/ERGO (eV)**	
**C1s**	284.7	284.5	284.7	C–C / C=C (sp^2 carbon of GC and ERGO)

**Binding Energy**	285.5	285.3	285.4	C-N
	288.4	288.3	288.3	O–C=O (carboxyl functional groups at ERGO edges)
**N1s Binding Energy**	399.7	399.8	399.9	C-N
	400	400.2	400	C-N
**O1s Binding Energy**	531.1	531.1	530.9	C=O (carbonyl oxygen from ERGO)
	531.7	531.7	531.5	C–O (hydroxyl/epoxy oxygen from ERGO)
	533.4	532.2	533.1	O–C=O (carboxyl oxygen from ERGO)
	535.7	-	535.6	Physically adsorbed water/oxygen (H_2O/O_2)

Table S6. Voltammetry responses for modified electrodes.

Electrodes	Ip_a (μA)	Ip_c (μA)	ΔE_p (mV)	Surface area (cm^2)
**Bare GC**	10.5	11.5	69	0.063
**GC/o-PDA**	9.9	10.8	71	-
**GC/m-PDA**	9.0	9.8	73	-
**GC/p-PDA**	7.8	8.7	87	-
**GC/o-PDA/GO**	9.9	10.98	70	0.0605
**GC/m-PDA/GO**	10.19	11.11	77	0.0613
**GC/p-PDA/GO**	10.4	11.48	78	0.0633

**GC/o-PDA/ERGO**	10.2	11.9	64	0.065
**GC/m-PDA/ERGO**	10.55	12.4	63	0.068
**GC/p-PDA/ERGO**	14.3	15.9	65	0.087

Table S7. Charge transfer resistance and electron transfer rate constant data of modified electrodes.

**Electrodes**	**R_{ct} (kΩ)**	**k_{et} (cm^2 s^{-1})**
**Bare GC**	21.3	1.78×10^{-3}
**GC/o-PDA**	29.9	1.27×10^{-3}
**GC/m-PDA**	34.8	1.09×10^{-3}
**GC/p-PDA**	42.8	9.3×10^{-4}

**GC/o-PDA/GO**	20.2	1.88×10^{-3}
**GC/m-PDA/GO**	19.2	1.98×10^{-3}
**GC/p-PDA/GO**	17.1	2.22×10^{-3}
**GC/o-PDA/ERGO**	14.5	2.62×10^{-3}
**GC/m-PDA/ERGO**	9.7	3.90×10^{-3}
**GC/p-PDA/ERGO**	7.9	4.81×10^{-3}

Table S8. Voltammetry responses obtained for electrocatalytic reduction of NBz at ArDAs ERGO electrodes.

**Electrodes**	**Potential (mV)**	**Reduction current (µA)**
**Bare GC**	-0.697	-26.3
**GC/p-PDA**	-0.709	-18.8

**GC/ERGO**	-0.69	-22.5
**GC/o-PDA/ERGO**	-0.675	-30.6
**GC/m-PDA/ERGO**	-0.651	-36.5
**GC/p-PDA/ERGO**	-0.612	-46.5

Table S9. Determination of NBz in river water sample using GC/p-PDA/ERGO.

[a] River water	Spiked (µM)	Found (µM)	Recovery (%)	RSD	Error (%)
Sample 1	10	9.9	99	1.01	-1
	80	79.9	99.8	0.071	-0.125
	150	149.9	99.9	0.038	-0.066

[a]Three replicate measurement
